# Cyclic stretch enhances reorientation and differentiation of 3-D culture model of human airway smooth muscle

**DOI:** 10.1016/j.bbrep.2018.09.003

**Published:** 2018-09-22

**Authors:** Shuichi Asano, Satoru Ito, Mika Morosawa, Kishio Furuya, Keiji Naruse, Masahiro Sokabe, Etsuro Yamaguchi, Yoshinori Hasegawa

**Affiliations:** a Department of Respiratory Medicine, Nagoya University Graduate School of Medicine, Nagoya 466-8550, Japan; b Department of Respiratory Medicine and Allergology, Aichi Medical University, Nagakute 480–1195, Japan; c Mechanobiology Laboratory, Nagoya University Graduate School of Medicine, Nagoya 466-8550, Japan; d Department of Physiology, Okayama University, Okayama 700-8530, Japan

**Keywords:** Asthma, Ca^2+^, Mechanotransduction, α-smooth muscle actin, Stretch, Tissue engineering

## Abstract

Activation of airway smooth muscle (ASM) cells plays a central role in the pathophysiology of asthma. Because ASM is an important therapeutic target in asthma, it is beneficial to develop bioengineered ASM models available for assessing physiological and biophysical properties of ASM cells. In the physiological condition *in vivo*, ASM cells are surrounded by extracellular matrix (ECM) and exposed to mechanical stresses such as cyclic stretch. We utilized a 3-D culture model of human ASM cells embedded in type-I collagen gel. We further examined the effects of cyclic mechanical stretch, which mimics tidal breathing, on cell orientation and expression of contractile proteins of ASM cells within the 3-D gel. ASM cells in type-I collagen exhibited a tissue-like structure with actin stress fiber formation and intracellular Ca^2+^ mobilization in response to methacholine. Uniaxial cyclic stretching enhanced alignment of nuclei and actin stress fibers of ASM cells. Moreover, expression of mRNAs for contractile proteins such as α-smooth muscle actin, calponin, myosin heavy chain 11, and transgelin of stretched ASM cells was significantly higher than that under the static condition. Our findings suggest that mechanical force and interaction with ECM affects development of the ASM tissue-like construct and differentiation to the contractile phenotype in a 3-D culture model.

## Highlights

• Airway smooth muscle (ASM) cells were embedded in collagen gel for a 3-D model.
• Cyclic stretch enhanced alignment of cell and F-actin.
• Cyclic stretch promoted differentiation to the contractile phenotype.

## 1 Introduction

Contraction of airway smooth muscle (ASM) plays a central role in airway narrowing in asthma. Increased ASM mass due to cell proliferation, hypertrophy, and migration is involved in the mechanism of pathophysiology of airway remodeling. Therefore, ASM is an important therapeutic target for airway diseases, specifically asthma and chronic obstructive pulmonary disease [1]. In order to uncover mechanisms underlying activation of ASM cells, two-dimensional (2-D) *in vitro* cultures of ASM cells have widely been used due to difficult availability of human ASM tissue samples [2], [3], [4]. However, ASM cells *in vivo* exist as a part of complex three-dimensional (3-D) structures with the extracellular matrix (ECM). Within the airway wall, ASM exists as an aligned population that wraps around the bronchiole in a helical fashion *in vivo* [5], [6]. Due to this unique arrangement, the angle of orientation and cell alignment are major factors that determine the phenotypes and properties of ASM cells [7]. Therefore, development of bioengineered 3-D models of ASM tissues is warranted to assess functional properties for pharmacological and biophysical studies [8], [9], [10].

The lungs and airways are continually exposed to mechanical forces such as shear stress, compression, and stretch during tidal breathing and pulmonary circulation *in vivo*. These mechanical stresses are involved in the mechanisms underlying the normal physiology and development of the respiratory system and pathogenesis of asthma [11]. In 2-D culture models of ASM cells, cyclic stretch induces cell alignment perpendicular to the stretch axis with reorganization of the cytoskeleton [12], [13]. However, the roles of mechanical stresses in the development of bioengineered 3-D models of ASM tissues are not known.

This study was designed to develop a 3-D model of ASM. For this purpose, human ASM cells were embedded in a collagen gel [9], [10]. We further examined the effects of cyclic mechanical stretch, which mimics tidal breathing, on the regulation of cell orientation, formation of stress fibers, and phenotype. We postulated that when cultured three-dimensionally within collagen gel with cyclic stretch, ASM cells develop tissue-like behavior by upregulating expression of genes for contractile proteins.

## 2 Materials and methods

### 2.1 Cells

Primary cultures of normal human bronchial smooth muscle cells from three different donors were obtained from Lonza (Walkersville, MD) and maintained in SmGM-2 culture medium (Lonza) containing 5% fetal bovine serum (FBS) in an atmosphere of 5% CO_2_ and 95% air at 37 °C [2], [13], [14]. Cells of passages 4–8 were used.

### 2.2 Fabrication of 3-D constructs

**Fig. 1:**
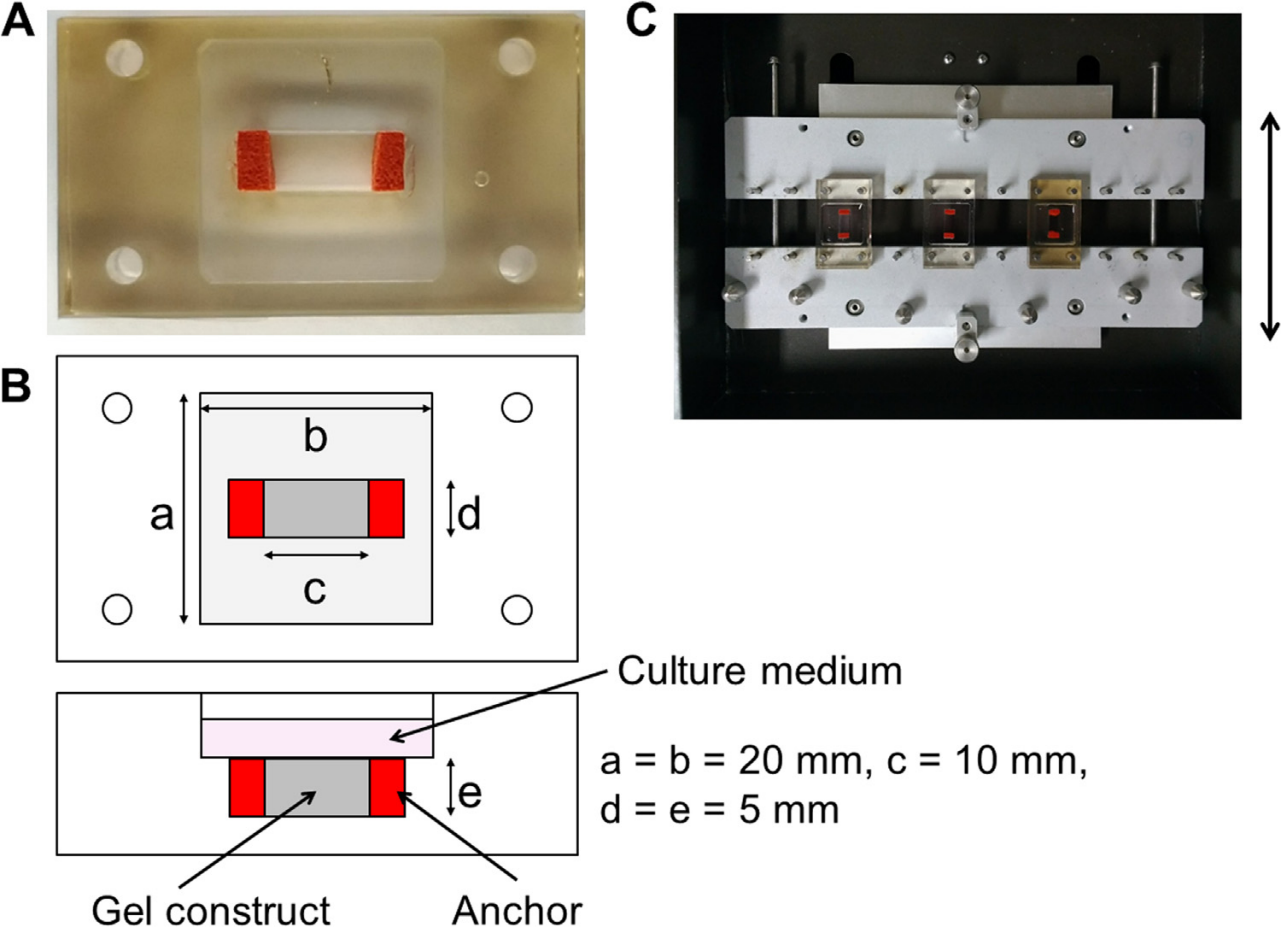
Stretching chamber and stretch device. (A) An overview of the silicone chamber in which human airway smooth muscle (ASM) cells are cultured within a type-I collagen construct. **(B)** A scheme of the silicone chamber. **(C)** An overview of the stretch device (ST-140; Strex) on which three silicone chambers are fit. The arrow indicates stretch direction.

ASM (5 ×10^5^/ml) cells were suspended in a solution of 2 mg/ml of type I collagen (Cellmatrix; Nitta Gelatin, Osaka, Japan) in SmGM-2 cell culture medium (Invitrogen, Carlsbad, CA) containing 5% FBS at room temperature. The solution was transferred into the well (10 mm in length x 5 mm in width x 5 mm in depth) of a silicone chamber with sponge anchors on both sides (STB-CH-3.5GS; Strex, Osaka, Japan) (Fig. 1**A and B**). The solution was able to infiltrate into the sponge. The gel was allowed to polymerize and attached to the sponge anchors by incubation at 37 °C for 15 min. After the gels were polymerized, 2 ml of SmGM-2 cell culture medium containing 5% FBS was added to the gel (Fig. 1**A**), then the medium was changed every other day.

### 2.3 Application of cyclic and static mechanical stretch

After the gel constructs were incubated for 24 h and polymerized, a uniaxial sinusoidal stretch of 12% strain at 30 cycle/min was applied for 48 h using a stretching apparatus driven by a computer-controlled stepping motor (ST-140; Strex) [2], [13], [15]. Briefly, one end of the chamber was attached to a fixed frame, while the other end was attached to a movable frame (Fig. 1**C**). The other two sides were free to move. The movable frame was connected to a motor driven shaft whose amplitude and frequency of stretch was controlled by a programmable microcomputer. Strain was calculated from the displacement of the silicone chamber before and after the stretch. Cells incubated under a static condition in the silicone chamber were used as a time-matched control.

### 2.4 Immunofluorescence staining

Cells grown within collagen gels were fixed with 4% formaldehyde for 30 min and permeabilized with 0.2% Triton X-100 (Sigma-Aldrich, St. Louis, MO) in PBS for 30 min. This was followed by blocking with 1% bovine serum albumin (BSA) in PBS for 60 min. Then, the cells were incubated with a mouse polyclonal anti-α-SMA antibody (dilution 1:400, a2547; Sigma-Aldrich) in PBS containing 1% BSA overnight, washed, and further incubated with a goat anti-mouse secondary antibody (dilution 1:1000, A-11001; Thermo Fisher Scientific) for 60 min at room temperature. Filamentous actin (F-actin) and nuclei were stained with rhodamine-phalloidin (dilution 1:1000, R415; Thermo Fisher Scientific) and 4,6-diamino-2-phenylindole (DAPI) (dilution 1:1000, D523; Dojin, Kumamoto, Japan) for 60 min at room temperature. Immunofluorescence images were obtained using an upright laser scanning confocal microscope (A1RMP; Nikon, Tokyo, Japan), with a × 25/1.2 NA Plan Apo violet-corrected water immersion objective [16], [17]. Images were obtained in 2 µm steps and up to 250 µm in depth.

### 2.5 Measurement of cell orientation

**Fig. S1:**
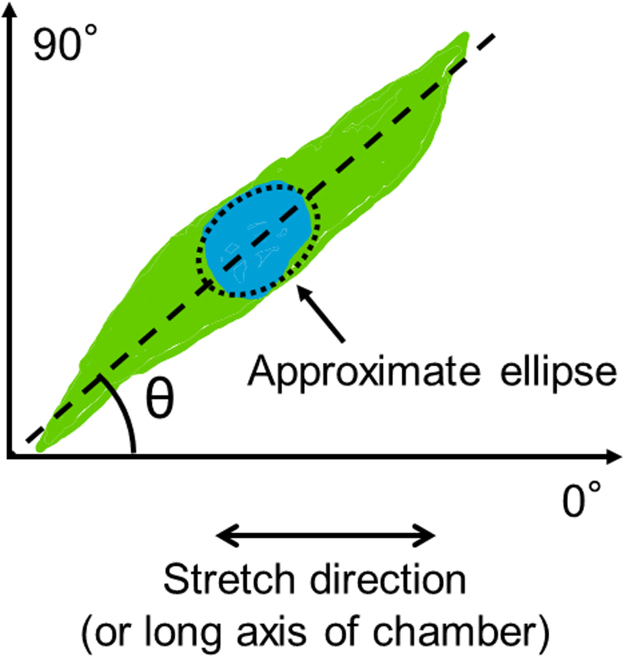
Supplementary Fig. S1. Schematic of how the angle of orientation of the long axis was measured. The angle (θ) is always between 0° and 90° with respect to the stretch direction or long axis of the chamber.

Images of the nuclei stained with DAPI were obtained using a confocal microscope with at least three arbitrarily selected visual fields. Optical volumes 517.6 µm x 517.6 µm x 200 µm were flattened into a single plane image. The orientation of each nucleus of the cell was measured as an angle (θ) of the long axis between 0° and 90° with respect to the stretch axis (Supplementary Fig. S1) using NIH ImageJ v1.33 software [13], [15].

### 2.6 Quantitative real-time PCR

Gels were immersed in liquid nitrogen and then minced. Total cellular RNA was extracted using Trizol reagent (Thermo Fisher Scientific, Waltham, MA). RNA was reverse transcribed to cDNA using a Superscript III kit (Invitrogen, Carlsbad, CA). TaqMan Gene Expression Assays for α-smooth muscle actin (α-SMA) *(ACTA2*) (Hs00426835_g1), calponin (*CNN1*) (Hs00959434_m1), myosin heavy chain 11 (*MYH11*) (Hs00975796_m1), transgelin (*TAGLN*) (Hs01038777_g1), and GAPDH (Hs99999905_m1) genes were purchased from Applied Biosystems (Foster City, CA). Quantitative polymerase chain reaction (PCR) amplification was performed on a 7300 Real-Time PCR system (Applied Biosystems) using the 3-stage program parameters provided by the manufacturer as follows: 2 min at 50 °C, 10 min at 95 °C, and then 40 cycles of 15 s at 95 °C and 1 min at 60 °C. Relative changes in mRNA expression compared to an unstimulated control and normalized to GAPDH were quantified by the 2^-ddCt^ method [2], [15].

### 2.7 Measurement of intracellular Ca^2+^ concentration

**Fig. S2:**
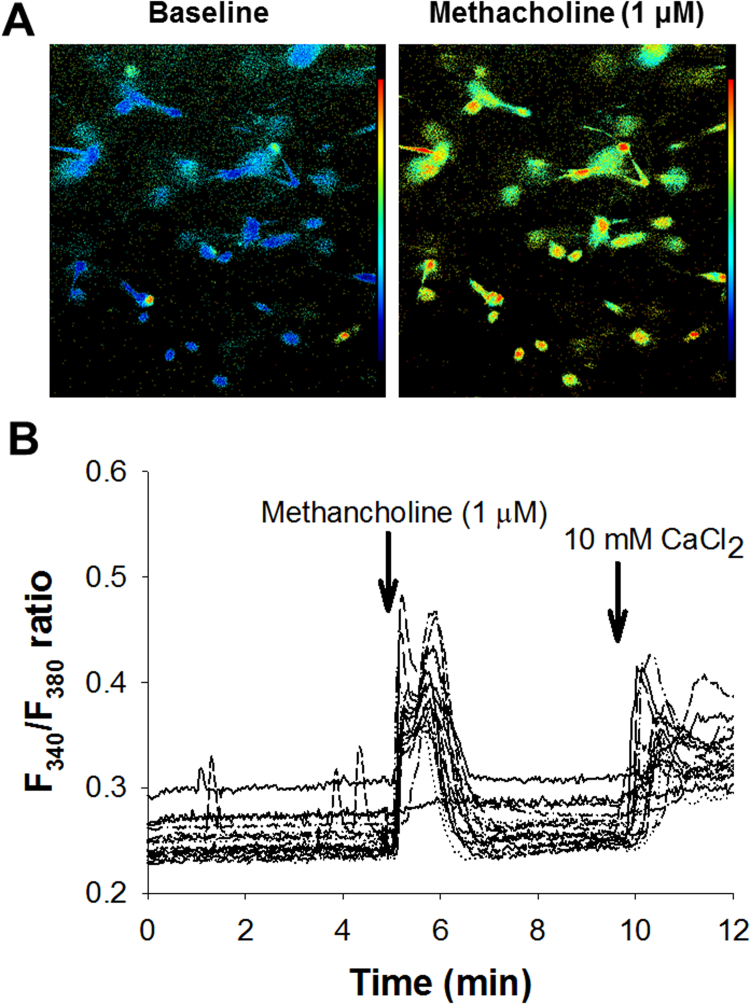
Supplementary Fig. S2. Elevation of intracellular Ca^**2+**^ concentrations in response to methacholine. (A) Representative images of changes in the fura-2 fluorescence signal, the ratio of F_340_ to F_380_ (F_340_/F_380_ ratio), an index of the intracellular Ca^2+^ concentration ([Ca^2+^]_i_), of human ASM cells embedded in collagen gel for 72 h before (baseline) and after application of 1 µM methacholine. The bright (red, yellow, and green) and dark (black and blue) colors represent higher and lower F_340_/F_380_ levels, respectively. **(B)** Typical time courses of the F_340_/F_380_ ratios of 17 cells for 1 µM methacholine (Sigma-Aldrich) and 10 mM CaCl_2_. The cells grown in a collagen gel under the static condition were treated with 5 µM fura-2/AM (Dojin) for 30 min at 37 °C in normal physiological solution containing (in mM): NaCl 145, KCl 5, CaCl_2_ 2, MgCl_2_ 1, glucose 10, and HEPES 10 (pH 7.40). After the cells were washed with normal physiological solution, the [Ca^2+^]_i_ was assessed by the fura-2 fluorescence using a fluorescence microscope (BX50WI; Olympus, Tokyo, Japan) with UMPlanFI 20 × /0.50 lens (Olympus) at room temperature. Data were analyzed using a digital fluorescence imaging system (Aquacosmos; Hamamatsu Photonics, Hamamatsu, Japan). The excitation wavelengths were set at 340 and 380 nm, and the emission was collected at 510 nm by a photomultiplier. The intensity of the fura-2 fluorescence due to excitation at 340 nm (F_340_) and 380 nm (F_380_) was measured after subtraction of the background fluorescence. See also **Supplementary Movie S2**.

Methods are described in Supplementary Fig. S2. Briefly, the cells grown in a collagen gel were treated with 5 µM acetoxymethyl ester of fura-2 (fura-2-AM) (Dojin). The intracellular Ca^2+^ concentration ([Ca^2+^]_i_) was assessed by the fura-2 fluorescence using a fluorescence microscope (BX50WI; Olympus, Tokyo, Japan). Data were analyzed using a digital fluorescence imaging system (Aquacosmos; Hamamatsu Photonics, Hamamatsu, Japan) [2], [18], [19].

### 2.8 Statistical analysis

Data are expressed as means ± standard deviation (SD). An unpaired *t*-test or analysis of variance (ANOVA) followed by Bonferroni's or Games-Howell's *post hoc* test was used to evaluate the statistical significance. *P* < 0.05 was considered statistically significant. Statistical analyses were performed using SPSS ver. 24 (SPSS Inc., Chicago, IL).

## 3 Results

### 3.1 Collagen gel and cells

**Fig. 2:**
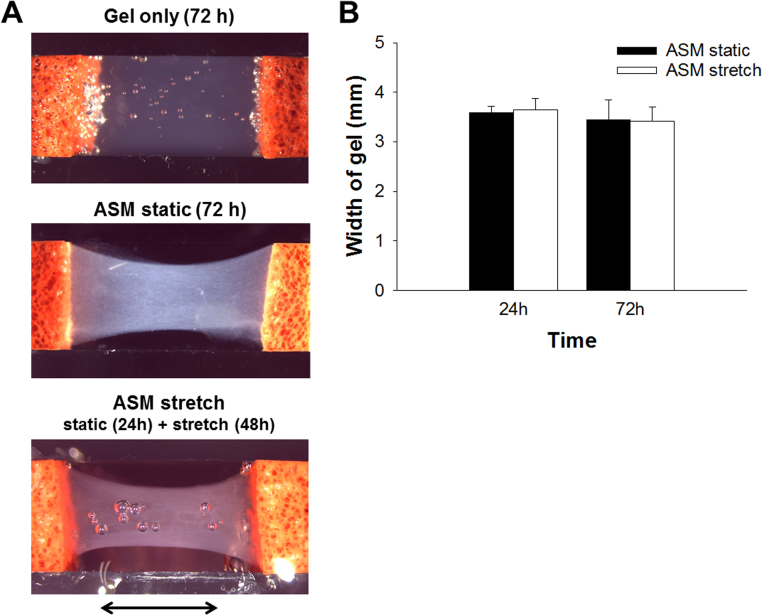
Gross appearance of gel constructs containing airway smooth muscle cells. (A) The type-I collagen gel (upper image) or gel containing ASM cells (middle image) was allowed to polymerize by incubation at 37 °C for 72 h under the static condition. 24 h after tissue fabrication under the static condition, cyclic stretch (12% in strain, 30 cycle/ minute) was applied to the gel containing ASM cells for 48 h (lower image). Arrow indicates stretch direction. **(B)** Time-dependent shortening of the width of gels (**d** direction in Fig. 1**B**) containing ASM cells under the static and stretched conditions are shown (n = 6).

Representative images of collagen gel and gel containing ASM cells after 72 h incubation in the silicone chamber are shown in Fig. 2**A**. Both edges of the gel were fixed to anchors. After 24 h incubation, the gel containing ASM cells was cyclically stretched or kept under the static condition for a further 48 h (Fig. 2**A**). The shape of collagen gel without cells was not changed by incubation for 72 h (Fig. 2**A**). In contrast, the width of the center of collagen gel constructs containing ASM cells was gradually shortened, indicating tension development due to cell contraction and collagen degradation. The width of the gel containing ASM cells became approximately 70% of the initial width 24 h after incubation (Fig. 2**A and B**). There was no significant difference between the width of the gels of the static and stretched tissues at 72 h (Fig. 2**B**).

### 3.2 Effects of cyclic uniaxial stretch on cell and F-actin alignment

**Fig. 3:**
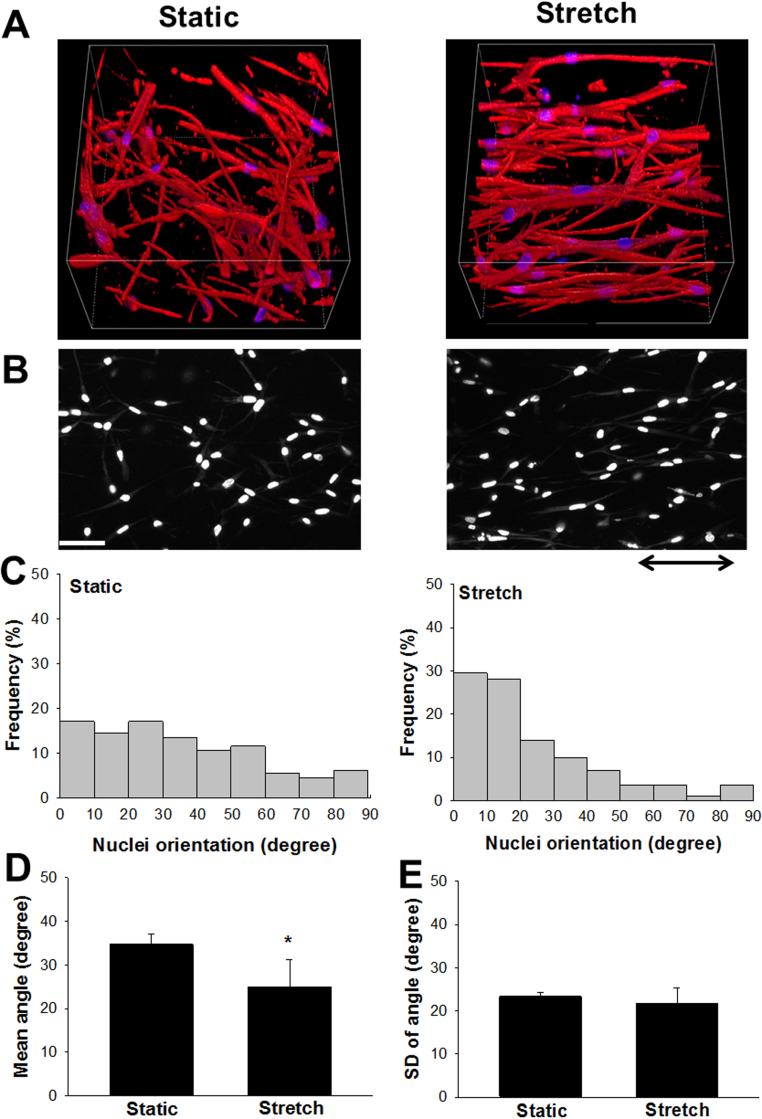
Effects of cyclic stretch on cell orientation in 3-D gel. (A) Representative 3-D rendered confocal fluorescence images stained for F-actin (red) and nuclei (blue) of ASM cells embedded in collagen gel with or without cyclic stretch. Z-stacks were acquired every 2 µm, and 3-D rendering was performed with NIS elements software using the Alfa-bending algorithm. Confocal images were obtained 72 h after fabrication. The 3-D image of stretched cells is also shown in **Supplementary Movie S1. (B)** 517.6 µm x 517.6 µm x 200 µm of optical volumes were flattened into a single plane image. Arrow indicates stretch direction. Bar = 50 µm. **(C)** Orientation of the ASM cells was assessed by angles of nuclei. Histograms of angles of nuclei divided into nine groups for every 10° of static (left) and stretched (right) cells are shown. The means **(D)** and standard deviations (SDs) **(E)** of the nuclei angles of static and stretched conditions were compared. Twenty-four hours after tissue fabrication, the gel was cyclically stretched (12% in strain, 30 cycle/minute) or kept under the static condition for a further 48 h. *Significantly different (*P* < 0.01) *vs.* the static condition (n = 4). Schematic of how the angle (θ) of orientation of the long axis was measured is shown in Supplementary Fig. S1.

Fig. 3**A** shows representative 3-D cell fluorescence images of F-actin and nuclei of the cells under the static and stretched conditions. The orientation of F-actin in static cells was relatively random. In contrast, F-actin in the cells that had been stretched within the gels for 48 h aligned along the direction of stretch (**Supplementary Movie S1**). To quantify the cell orientation, 517.6 µm x 517.6 µm x 200 µm optical volumes of static and stretched gels were flattened into a single plane image (Fig. 3**B**). Then, the orientation of each nucleus of the cell was measured. Histograms of cell orientation under the static and stretched conditions are shown in Fig. 3**C**. The average angles of the stretched cells were significantly smaller than those of static cells (Fig. 3**D**). The SD values, a measure of heterogeneity of cell direction [13], were not significantly different between the groups (Fig. 3**E**).

Supplementary material related to this article can be found online at doi:10.1016/j.bbrep.2018.09.003.

The following is the Supplementary material related to this article Movie S1.

Movie S1 **Effects of cyclic stretch on orientation of F-actin and nuclei in 3-D gel.** A movie of 3-D rendered confocal fluorescence images stained for F-actin (red) and nuclei (blue) of ASM cells embedded in collagen gel with cyclic stretch. Cyclic stretch was started 24 h after tissue fabrication then applied for 48 h. The 3-D image of stretched cells is also shown in **Supplementary Figure 3A,*****right***.

### 3.3 Effects of cyclic stretch on expression of contractile proteins

**Fig. 4:**
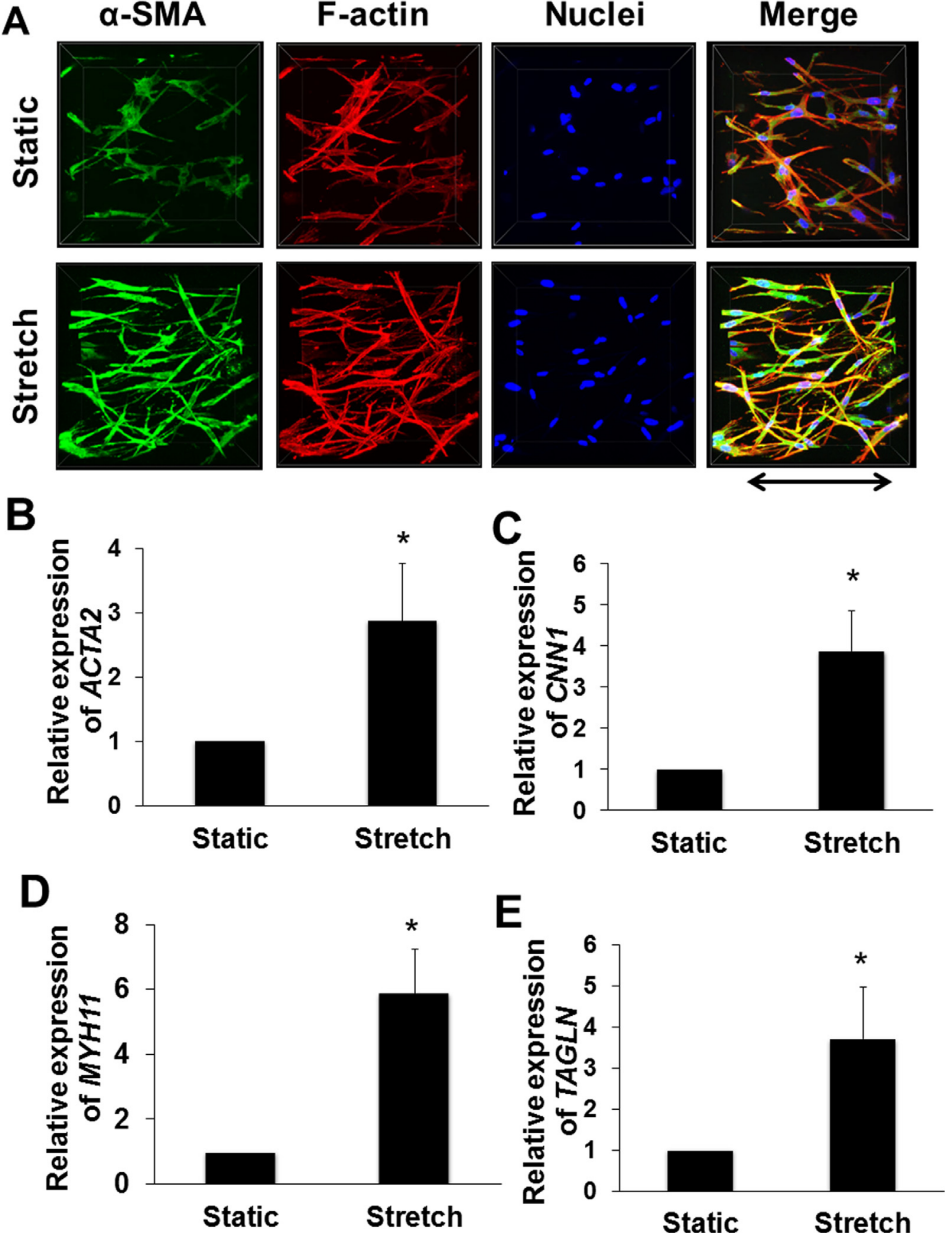
Effects of cyclic stretch on F-actin formation and expression of contractile proteins. (A) Representative 3-D immunofluorescence images of ASM cells cultured within gels with or without cyclic stretch. Reconstructed Z-stack confocal images of collagen gel constructs stained with α-smooth muscle actin (α-SMA), F-actin, DAPI, and merged images. Arrow indicates stretch direction. Effects of cyclic stretch on mRNA expression of α-SMA (*ACTA2*) **(B)**, calponin (*CNN1*) **(C)**, myosin heavy chain 11 (*MYH11*) **(D)**, and transgelin (*TAGLN*) **(E)**. Cyclic stretch was started 24 h after tissue fabrication and then applied for 48 h. Data are mean ± SD (n = 6). *Significantly different (P < 0.05) *vs.* the static condition.

We investigated whether cyclic stretch induces differentiation of ASM cells to the contractile phenotype. Expression of α-SMA protein was used as an indicator of a contractile ASM phenotype. Fig. 4**A** shows immunofluorescent images of α-SMA-positive stress fibers, fluorescent F-actin, and merged images of ASM cells cultured within collagen gels with or without cyclic stretch. α-SMA-positive stress fibers were increased in the stretched cells. Real-time quantitative PCR data show that mRNA levels of contractile protein genes, α-SMA (*ACTA2*), calponin (*CNN1*), myosin heavy chain 11 (*MYH11*), and transgelin (*TAGLN*), in the stretched cells were significantly higher than those in the static cells (Fig. 4**B**). In our preliminary experiments, relative mRNA levels of *ACTA2*, *CNN1*, *MYH11*, and *TAGLN* in ASM cells cultured on the 2-D plastic dish to those in the 3-D gel under the static condition were 1.68, 4.03, 0.97, and 7.67, respectively.

### 3.4 Elevation of intracellular Ca^2+^ concentration by methacholine

Next, we examined whether the ASM cells cultured within the collagen gel for 48 h under the static condition exhibited elevation of [Ca^2+^]_i_ in response to methacholine. Supplementary Fig. S2A shows representative cell images of the F_340_/F_380_ ratio, a measure of [Ca^2+^]_i_, before and after the application of 1 µM methacholine. In the visualized field, most of the cells exhibited elevated F_340_/F_380_ ratios in response to methacholine Time courses of the changes in the F_340_/F_380_ ratio of the cells in response to 1 µM methacholine and a high concentration (10 mM) of CaCl_2_ are show in Supplementary Fig. S2B. The changes in [Ca^2+^]_i_ are also shown in **Supplementary Movie S2**.

Supplementary material related to this article can be found online at doi:10.1016/j.bbrep.2018.09.003.

The following is the Supplementary material related to this article Movie S2.

Movie S2 **Elevation of intracellular Ca**^**2+**^**concentrations in response to methacholine.** A movie of changes in the fura-2 fluorescence signal, F_340_/F_380_ ratio, an index of [Ca^2+^]_i_, of human ASM cells embedded in collagen gel for 72 h. The cells were stimulated by 1 µM methacholine and then by 10 mM CaCl_2_. The bright (red, yellow, and green) and dark (black and blue) colors represent higher and lower F_340_/F_380_ levels, respectively. See also Supplementary Fig. S2.

## 4 Discussion

The main findings of the present study are that in a 3-D culture model of ASM: (1) culture of human ASM cells within type-I collagen exhibited a tissue-like structure with F-actin formation, (2) uniaxial cyclic stretch enhanced alignment of nuclei and actin stress fibers, and (3) expression of mRNAs for contractile proteins such as α-SMA, calponin, myosin heavy chain 11, and transgelin of stretched ASM cells was significantly higher than that within the static gel. To our knowledge, we demonstrated for the first time that cyclic stretching enhanced cell reorientation with α-SMA expression in the 3-D model of ASM.

It is well-known that the cells in 2-D cultures distribute randomly under the static condition without stretching [12], [13], [15], [20], [21]. Interestingly, actin stress fibers of ASM cells slowly tend to align parallel to the stretch direction without applying cyclic stretch in our 3-D models. The average angle of the cells becomes closer to 45° when the distribution of cell orientations is random [13], but the average nucleus angle of static cells was approximately 35° under the static condition for 72 h (Fig. 3**B**). In our method, both ends of the 3-D gel were attached to sponge anchors, and the time-dependent shortening was observed in the gel containing ASM cells but not in that without cells, indicating tension development due to cell contraction and cell-ECM interaction (Fig. 2). Similar to our results, West et al. reported that when both ends of 3-D tissues, in which ASM cells together with NIH3T3 fibroblasts are cultured within collagen gels, are fixed, ASM cells tend to align along with the long axis with tension formation [9]. Therefore, the difference in cell orientation under the static condition between 2-D and 3-D models possibly derives from the directed intrinsic tension formation of the cells within the gels and mechanical interaction with surrounding ECM [21].

Cell reorientation toward the stretch direction was enhanced by cyclic stretch in the present 3-D culture of ASM cells (Fig. 3). Using a 2-D flat cell culture system, we and other groups demonstrated that cyclic stretch induces cell alignment perpendicular to the stretch direction in various cell types [12], [13], [15], [20], [21]. The difference in direction of alignment is in good agreement with the findings in vascular smooth muscle cells [21], [22]. It is considered that the cells change their orientation to minimize the intracellular stress and cellular damage in 2-D cell cultures [21]. In contrast, when smooth muscle cells are embedded within the 3-D gel, the cells mechanically interact with the surrounding cells and ECM, leading to alignment along the stretch direction. However, due to the limitation of our imaging system, a distribution on cell density and orientation could not be acquired across the whole gel. Therefore, involvement of heterogeneity in cell density and orientation within a gel cannot be excluded. Another important issue is that cell migration within the gel might contribute to cell alignment and morphological change. Future studies and improvement of the present system are necessary.

We demonstrated that the expression of α-SMA protein and mRNAs for contractile proteins increased with the cyclic stretching (Fig. 4). Similar results were reported in a 3-D model of vascular smooth muscle [21]. Acquisition or increased expression of α-SMA, one of the six known eukaryotic actin isoforms, characterizes differentiation from a proliferative-to-contractile phenotype and increased contractile ability of ASM cells [23], [24], [25]. It is known that concentrations of contractile proteins decrease when ASM cells are cultured and passaged under the static 2-D condition [4], [25]. Moreover, substrate stiffness regulates cellular properties and expression of contractile proteins in various cell types including ASM cells [17], [26], [27]. Our findings suggest that activation of cellular mechanotransduction is involved in the mechanisms of differentiation and expression of genes for contractile proteins induced by cyclic stretch in 3-D culture of ASM cells embedded in the collagen gel.

In the present study, a 12% uniaxial cyclic strain at 30 cycle/min was applied to the gels in accordance with methods described in our previous reports using 2-D culture of ASM cells [2]. The expected physiological range of tidal muscle stretch during breathing is from approximately 4% of muscle length during spontaneous breathing at rest to 12% during a sigh [28]. We previously demonstrated that human ASM cells release ATP in response to 12% uniaxial stretch (30 cycle/min for 15 min) [2]. Moreover, a single 10% stretch induces [Ca^2+^]_i_ elevation *via* activating stretch-activated channels in 2-D culture of ASM cells [18]. Taken together, the sinusoidal stretch protocol (12% strain at 30 cycle/min) is within a physiological range and mimics the physical and biological properties of the airway wall.

High-throughput screening for drugs modulating contractile forces of ASM is beneficial to find novel therapeutic strategy and to understand the pathogenesis of asthma. Therefore, one of the goals of developing bioengineered ASM cells and tissues is to measure physiological and biophysical properties [3], [8], [9], [24], [29]. In our model, ASM cells within the collagen gel exhibited intracellular Ca^2+^ mobilization in response to methacholine (Supplementary Fig. S2) as seen in intact tissues [30]. The increase of [Ca^2+^]_i_ plays a pivotal role in activation specifically of contraction of ASM cells [31]. Nesmith et al. designed and built an *in vitro* model of human ASM tissue and measured contraction [29]. Park et al., 2-D cultured ASM cells on polyacrylamide-based gel substrates and measured the contractile force of each ASM cell using Fourier-transform traction microscopy [3]. Future studies are necessary to extend our 3-D model and develop the system to assess its contractile force as well as [Ca^2+^]_i_.

In summary, we developed an engineered ASM tissue-like construct that exhibits characteristics of ASM such as F-actin alignment, α-SMA expression, and elevation of [Ca^2+^]_i_ in response to methacholine using a 3-D culture of ASM cells within a collagen gel. Furthermore, cyclic mechanical stretch enhanced differentiation to the contractile phenotype. Our findings suggest that mechanical forces, both intrinsic tension formation and externally applied cyclic stretch mimicking tidal breathing, play an important role in development of ASM tissue-like behavior in a 3-D culture model.
